# Applying the ‘Candidacy’ Model to understand access to key nutrition, food & health services in LMIC contexts: a qualitative study in Odisha, India

**DOI:** 10.1007/s12571-023-01357-5

**Published:** 2023-04-04

**Authors:** Rebecca Mitchell, Jessica Gordon, Gopal Krushna Bhoi, Nicholas Nisbett

**Affiliations:** 1 Institute of Development Studies, Brighton, UK; 2 DCor Consulting, Bhubaneswar, Odisha India

**Keywords:** Candidacy, Food security, Health, Nutrition, India, Access, Barriers

## Abstract

In order to make progress towards Sustainable Development Goal 2 – Zero Hunger - we must acquire a better understanding of what continues to hamper achieving food security, particularly in contexts where progress has been achieved, but has then faltered. This article investigates access to nutrition and food services in three of the Indian state of Odisha’s traditionally poorer districts, where a large number of the state’s most marginalised populations live. Semi-structured interviews were carried out in 11 villages. The Dixon-Woods Candidacy Model was employed to provide greater insight into the experiences of access to health and nutrition services, from both the supply and the demand sides. We found that there are many points along the journey that hamper access. We identified two levels of gatekeepers that can create (or remove) barriers, the first as front-line service providers and the second as high-level officials. The candidacy model shows that marginalisation caused by identity, poverty and education disparities hampers progress throughout this journey. This article aims to provide a view to improve our understanding of access to health, food and nutrition services, to improve food security, and to show the value of the candidacy model applied to an LMIC health setting.

## Introduction

Availability of community level food, nutrition and health services, particularly for marginalised populations, is a key priority within the global health agenda. Efforts to improve global food security and nutrition were reinvigorated by the Sustainable Development Goals with a commitment to achieve Zero Hunger and Universal Health Coverage by 2030. Malnutrition is recognised as a social and political problem, with solutions that should be framed accordingly (Gillespie et al., 2013; Guthman, 2014; Nichols et al., 2022). A number of frameworks that focus on barriers and enablers to accessing health and nutrition services have been developed to guide research and policy, but a criticism has emerged that these frameworks focus on formal points of access and utilisation without full attention to the actual experience of service seekers (Kelner & Wellman, 1997; Dixon-Woods et al., 2005). The concept of candidacy was created to reflect the broader experience of access to health care, to explore self-awareness of medical needs, knowledge of services and the effort required to access services, particularly in relation to those who control access, the gatekeepers of services (Dixon-Woods et al., 2005, 2006), and incorporates the social and political context of service users. These inquiries are especially relevant in low and middle income countries (LMIC) where services are in short supply, and where marginalisation and discrimination based on, e.g. gender, caste, or disability, restricts access, and reduces the ability to negotiate with formal or informal service providers (Joshi, 2006; Pradhan & Roy, 2019). This study therefore applies the candidacy framework to understand the journey that users of government-provided food, nutrition and health services undertake in Odisha, India. As such, this is one of the first applications of the candidacy model to food security and health systems strengthening objectives in an LMIC. This article is thus a useful accompaniment to emerging critical nutrition literature but operationalises such perspectives in the context of access to health and nutrition services, using a model established in the context of social inclusion.

Despite the economic growth India has experienced in recent decades, incidence of undernutrition and related childhood illnesses remain high, particularly amongst the rural poor (Sandhu, 2014). India ranked poorly compared its neighbours on the 2019 Global Hunger Index at 102 out of 117 qualifying countries; Nepal ranks 73^rd^, Bangladesh 88^th^, and Pakistan 94^th^ (von Grebmer et al., 2019). In 2019 the prevalence of stunting in children under five years was 37.9% and wasting 20.8%, and the proportion of the overall population that was undernourished was 14.5% (von Grebmer et al., 2019). Undernutrition is known to lead to poorer health outcomes and lower educational performance amongst children and is estimated to account for as much as 45% of all deaths of children under five as an underlying cause (Black et al., 2013). As a result, India has been strengthening and enlarging its safety nets, thanks in part to lobbying by civil society activists in the early 2000s, leading to a National Food Security Act (NFSA), ratified in 2013. The aim of the NFSA is to “ensure access to adequate quantity of quality food at affordable prices” (GOI, 2013) by bringing together four pre-existing health and nutrition programmes. These are the Targeted Public Distribution System (TPDS), Integrated Child Development Services (ICDS), the Midday Meal programme (MDM) and the Indira Gandhi Matritva Sahyog Yojana maternity benefit programme, referred to as MAMATA in Odisha (Puri, 2017).

The four NFSA services can be summarised as follows; TPDS is a food distribution scheme that targets food-insecure households. MDM is a school-based feeding programme targeting children aged 6–14 years. ICDS is a broader programme that provides supplementary feeding for children aged 6 months to 6 years and pregnant and lactating women, as well as education and counselling services, weighing and growth monitoring of children, immunisation, health checks and referral services for its target population. MAMATA is a conditional cash transfer scheme providing financial support to pregnant and lactating women to enable them to seek improved nutrition and promote health seeking behaviour (Gordon et al., 2019). The NFSA was designed to reduce food insecurity and improve nutrition; however stunting and wasting persist at the high levels. Understanding the obstacles to NFSA services is necessary to improve nutrition and may offer insights for nutrition improvement elsewhere.

The state of Odisha has extremely high levels of undernutrition. Despite the introduction of the NFSA, child wasting (weight for height) has increased from 19.6% to 20.4% and the proportion of women with anaemia has increased from 51%  to 61.1% between 2005–6 and 2015/16 (IIPS, 2016). Recognising the issues, the Government of India (GOI) has set high targets for the numbers of households it expects the state government to reach with NFSA programmes; 82% of rural households and 55% of urban households (based on 2011–12 National Sample Survey Office (NSSO) data), which is much higher than the national average (GOI, 2013). The most recent census (2011) showed that 83% of the state population live in rural areas, with agriculture as primary occupation. 23% of the population of Odisha belong to Scheduled Tribes (ST) (vs. 8.2% for the whole of India), and 16% belong to Scheduled Castes (SC) (as the whole of India). Odisha has an estimated poverty rate of 33% (vs. 22% for the whole of India) with considerable social and spatial variation across the state; rural poverty rate in the most backward districts is 68% vs. 46.9% for the state rural average (Thomas et al., 2015). ST and SC populations are the most marginalised and the poorest in India (Diwaker, 2014; Mosse, 2018) who are therefore expected to experience worse health and nutrition outcomes than the rest of the population (Barros et al., 2010; Nisbett et al., 2022).

Castes in India are divided into categories by the Government of India for administrative purposes (John et al., 2020). Because these categories are used for forms of positive discrimination in education and jobs they also reflect traditional hierarchies: traditionally ‘high-caste’ groups such as Brahmins are in the ‘General Caste’ category (sometimes known also as ‘Forward Castes’) and those who were traditionally the most excluded and repressed, in the ‘Scheduled Caste’ group or, if they belong to one of India’s Indigenous or Adivasi Peoples, the ‘Scheduled Tribe’ group. They are often simply known by their acronyms, SC and ST. Other castes not in the general caste category belong to a category referred to as ‘Other Backward Castes’ or ‘OBC’. Although caste inequality has reduced in some states in India, particularly the south (Chakrabarti, 2021), caste discrimination is still rife in many contexts, including in health and nutrition services (John, 2018; Mosse, 2018; Deshpande, 2019), and in Odisha, those belonging to ST and SC groups experience worse outcomes compared to other caste categories as shown by recent National Family Health Survey five (NFHS V) data (IIPS, 2021). The literacy rate of ST and SC groups in Odisha is 52.2% and 69% respectively, well below that of the total for the state 72.9% (NFHS V) and poverty rates for ST and SC populations remain extremely high, with ST groups at 63% and SC groups at 41% compared with the state level 33% (Panda & Padhi, 2021). The districts that were part of this study, known as the ‘KBK + ’ districts (pertaining to the older boundaries of Kalahandi, Koraput and Balangir, now divided amongst several other districts), are known to be amongst the most deprived in Odisha and in India. The combination of a rural, marginalised, poor population has meant that Odisha has multiple intersecting barriers to overcome in order to successfully deliver NFSA services to its whole population.

Despite these challenges, Odisha has seen some recent improvements in health and nutrition outcomes. The infant mortality rate has declined from 65% in 2005–6 to 40% in 2015–16, and the rate of stunting in children under five years (height for age) has declined from 45% to 34.1% over this same period. Recent research has shown some improvements in health service coverage and associated determinants of child nutrition, such as increased exclusive breastfeeding and weaning at the World Health Organisation (WHO) recommended time (Avula et al., 2015; Kohli et al., 2017). However, these improvements have not led to better health and nutrition *outcomes* for all. As noted above, child wasting levels have *increased* and major inequalities in nutrition outcomes still exist based on socio-economic status, caste, gender and geography. Nutrition improvements need specific nutrition services to be delivered alongside health and food support for significant change to be achieved (Kohli et al., 2017). Understanding the barriers to access to NFSA in Odisha will provide evidence to inform these improvements.

This article investigates access to nutrition, food and health services in some of Odisha’s poorer districts, where many marginalised ST populations reside. We begin by providing an overview of the research context and of the Candidacy Model. Results are then presented and discussed with a view to improve our knowledge of access to health, food and nutrition services and the relevance of the candidacy model to a LMIC health setting.

### Research context and research question

This study was part of a project evaluation that assessed the use of social audits to improve the delivery of NFSA programmes. The social audit programme included education about NFSA entitlements and grievance redressal mechanisms, while simultaneously auditing programmes. The evaluation found that social audits did result in some improved knowledge of entitlements and forms of participation, and some evidence of improved access (Gordon et al., 2019). The qualitative interviews that form the basis of this article were undertaken during the social audit intervention. The social audit itself is not immaterial to the discussion, however. Here we aim not to reflect on the success of the social audit model, but to understand the wider process of access to NFSA programmes through the use of the candidacy model, acknowledging that some of these processes will have been affected by the social audit. Understanding the barriers that persist despite the social audit process, is particularly relevant for contexts where similar programmes continue, or are being considered. Our research question is therefore: to what extent do various factors of candidacy help explain the barriers to, and the experience of nutrition, food and health support services in marginalised districts of Odisha?

Research was carried out within 11 villages in three districts of Odisha. Community members were interviewed by local researchers about their experiences in accessing NFSA services, as well as their experience of the social audit programme. We sought to understand why NFSA programmes were failing to reach the most vulnerable.

### Framing the concept of candidacy

The concept of candidacy (or entitlement) for services was generated to assess both healthcare equitability and access in the UK, as an alternative to the measure of utilisation which focuses on access alone (Dixon-Woods et al., 2005, 2006). It recognises that accessing a service is a negotiation between potential users, and gatekeepers and providers, and these interactions are key to whether access is achieved (Dixon-Woods et al., 2006). It has been used effectively to analyse access to other public services, and applied successfully to vulnerable and marginalised groups (Manthorpe et al., 2009; Koehn, 2009; D’Ambruoso et al., 2010; Mackenzie et al., 2013; Thomas et al., 2019), within a variety of settings, including the UK (Dixon-Woods et al., 2005, 2006; Garret et al., 2012), Australia (Peiris et al., 2012) Canada (Koehn, 2009) and South Africa (Adeagbo et al., 2019). The model will be applied to NFSA service access in Odisha.

There are seven stages in the Dixon-Woods et al. (2005) Candidacy Model. 1) Identification by an individual (or household) of candidacy (eligibility) for a service, or self-awareness of needing a service. 2) Navigation of services, whether an individual has knowledge of a)the existence and b)availability of services. 3) Permeability of services, what demands are made by supply-side gatekeepers and service providers for individuals to show they qualify for treatment. 4) Asserting candidacy at services, how much work is required by individuals seeking a service (demand-side users), when interacting with service providers. 5) Adjudication by professionals, does the service provider or gatekeeper consider an individual legitimate in their claim of eligibility. 6) Use or resistance to services, whether individuals who are offered access to services decide to use it. 7) Local operating conditions, factors that influence decisions about service provision and use, such as perceived or actual availability of services and resources, and relationships that may develop over time between providers and recipients.

Alternative conceptual frameworks that identify barriers to access to health and nutrition services specifically within LMIC contexts find several themes that are relevant to our study (Ensor & Cooper, 2004; Peters et al., 2008; Jacobs et al., 2012). Geography, availability and acceptability of services provide a grounding in two of the frameworks (Peters et al., 2008; Jacobs et al., 2012), with supply and demand side issues cutting across all three (Ensor & Cooper, 2004). However, these frameworks focus on whether utilisation of services was achieved along formal points of access, and unlike the candidacy model do not explore informal factors and interactions that take place which may affect why and how access was realised, or not. These frameworks do include analysis of the social and economic background of service users, but this is framed in terms of discrete supply and demand issues such as the direct costs of access or delivery, and poor health education and awareness. The candidacy model provides opportunity to incorporate all these key issues and explore how they influence access to NFSA services.

Geographic barriers (e.g. remoteness, physical barriers, lack of access roads) influence local operating conditions (stage seven of the candidacy model) and affect whether eligible individuals can use services (stage six). One of the goals of the NFSA is to reach the most remote and marginalised people, by overcoming geographic barriers. Availability of services is also affected by local operating conditions. These conditions include supply chain problems (Tanksale, 2015; Tripathy, 2011), whether households have knowledge of NFSA services (stage two), whether individuals feel able to assert their candidacy (stage four), and whether they want to use the services offered (stage six). Acceptability of services is again affected by the local operating conditions, including the quality of service and competence of staff (Peters et al., 2008; Jacobs et al., 2012).

Therefore the concept of candidacy is useful in this context for three central reasons. Firstly, it offers an opportunity to see how factors that relate to the supply of NFSA services and demand for those services interact, rather than viewing them as two separate issues. Secondly, it is particularly relevant in contexts where marginalisation and discrimination based on individual or group identity, or other social determinants of health (SDH), may restrict access to services. Candidacy is affected by identity, education and subsequent interactions with gatekeepers and providers. And thirdly, because it goes beyond just understanding whether a service is used, it provides the opportunity to explore the issues and barriers that underlie utilisation, such as cultural misunderstandings, gatekeepers, and institutionalised discrimination, amongst others (Dixon-Woods et al., 2006). The model offers an opportunity to view NFSA service use as a journey, understanding that there are multiple points and perspectives along that journey that prevent access, and that relationships and communication between users and providers is key to whether candidacy is recognised and access achieved.

The socio-economic background of individuals seeking health and nutrition care will affect their ability to recognise the candidacy for services (stage one), their knowledge of services and ability to physically access them (stage two), and it may influence whether they feel able to assert candidacy (stage four). The expectations and preferences of service users are also affected by socio-economic conditions including levels of education, their community and culture, and previous experience with the service (WHO, 2008; Marmot et al., 2010; Jacobs et al., 2012). The NFSA is required to deliver services to a widely diverse population, with widely diverse expectations and needs (Kohli et al., 2017). Each of these issues is affected by pervasive poverty and marginalisation of the target population (Peters et al., 2008; Sharma et al., 2009; Jacobs et al., 2012; Tanksale, 2015; Kohli et al., 2017).

## Methods

Qualitative research was undertaken as part of a wider mixed-methods design, which included a quantitative survey and process evaluation. The qualitative component explicitly explored community level perceptions and contextual factors that may enhance or inhibit access to NFSA services. We applied a multi-site case study approach to gather detailed descriptive community level data from a sample of 11 villages in three districts of Odisha. The villages were purposively chosen based on distance from the district capital (close to, medium distance, and far from capital). Participants were then purposively chosen to achieve a mix of gender, ST, SC and OBC, locations within the village (based on distance from the centre), and to include those who did and did not participate in the social audit. There was no intention to create statistical significance, but rather provide rich contextual insights from a variety of perspectives where we had purposively selected people to include a gender balance and to cover marginalised groups extensively. The data used in this article is drawn from 82 semi-structured interviews. Forty men and 42 women were included, with an age range of 18–69. 24 participants identified as OBC, 29 as ST, and 27 as SC (two were not assigned to these categories), meaning that all bar two participants in this study were from the caste or tribal groups that are considered the most marginalised in comparison to those of ‘general’ or ‘forward caste’ status.

The research team was made up of three researchers from IDS, working alongside professional enumerators from DCor, a research consultancy firm based in Odisha. All researchers had experience in evaluating nutrition and health service access in India. The team worked collaboratively throughout the primary research phase of the project. The research team from DCor plan to revisit villages to share research findings, but this has been hindered by COVID-19 lockdowns.

Research protocols were developed by the whole team and crosschecked with quantitative tools and data to ensure complementarity. Pilot testing and training was carried out to ensure the tools were appropriate. The DCor team collected detailed field notes and carried out the interviews in the local language of the participants. Where possible the gender of the researcher was matched with the gender of the respondent. Interviews were carried out either within local community settings, such as the Anganwadi Centre, or participants homes, depending on their preference, or to avoid non-participant interruption. Non-participants were asked to leave to ensure privacy of answers, although within homes there were inevitably interruptions from partners or children. Participants were approached face to face, and asked to participate in the research. A small number of potential participants refused due to a lack of time. When this happened the researchers either returned to visit that participant at a mutually agreed on time, or looked for other participants with similar characteristics. No repeat interviews were carried out in this study.

Interviews were digitally recorded, downloaded, and anonymized daily. Recorded files were translated and transcribed verbatim into English. Transcripts were quality assured by senior researchers and coded using qualitative data analysis software in a two-stage process. The first level of coding used a scheme developed by the IDS team, based on the interview protocol themes. Themes included whether or not participants were able to access services, what prevented access, whether they participated in the social audit process, and whether they participated in community level governance mechanisms such as committees. Secondary coding included the application of the seven stage candidacy framework as described above, as well as deriving new codes from emergent themes within the data.

Ethical approval was granted by a local Institutional Ethics Committee in Odisha and by the author’s institutional ethics committee. The ethical application was led by IDS in collaboration with the Azim Premji Philanthropic Initiative (APPI) India and DCor. Consent forms were translated into local languages and verbally discussed. If participants were not able to read, verbal consent was obtained. Participants received a small gift as compensation for their time.

## Results

Of the 82 people interviewed, 79 were accessing one or more NFSA service through at least one family member. Over half (50) of the people interviewed identified specific barriers to accessing NFSA services, of these 15 were ST, 16 were SC and 18 were OBC (1 was unknown). 23 were male and 27 were female.

The following sections describe respondent’s experiences of accessing NFSA services with results grouped under the stages of the Candidacy model.

### Identification by an individual (or household) of candidacy for an NFSA service

This stage of the candidacy model asks whether individuals or households can identify their candidacy for NFSA services; do they recognise a need to use and access these programmes (Dixon-Woods et al., 2005). Some participants did identify candidacy for NFSA services by recognising a need to feed their hungry families. However, others relied on the NFSA criteria, such as possession of a ration card, or pregnancy. NFSA criteria assume that those eligible due to poverty, vulnerability, disability or age, will automatically have a nutritional and food security need (GOI, 2013), removing the individual agency of service users and placing the burden of responsibility of identifying candidacy on service providers.

#### TPDS

Ration cards are provided to individuals and households who have been identified as being a priority household or the ‘poorest of the poor’; this is measured by household income (Gordon et al., 2019). Individuals identified their candidacy for the programme largely based on eligibility for a ration card. However, some respondents reported that they recognised eligibility for a ration card, and therefore TPDS, but had not been able to access the ration card. A smaller number of respondents identified their eligibility due to a food security need (hunger), however, one respondent stated that being hungry is considered shameful, and not openly discussed, for ST people in particular, so there may be a hidden food security need.

#### MAMATA

There were many examples of community members who were unable to recognise candidacy for MAMATA benefits. Despite the presence in the family of a pregnant or lactating woman, when asked if they were aware of the benefit some respondents said ‘no’, whilst others had tried to access it despite not being eligible. This 58 year old OBC lady was frustrated that her daughter was not able to access MAMATA payments for her last baby, but her daughter had 5 children and so was not entitled,

“*Interviewer: Are you facing difficulties for MAMATA scheme?*

*Respondent: Yes, we are facing problems. When we go to ask for the financial grants they just say that it has not yet arrived even after a year. It has not arrived today come tomorrow and that goes on.*

*Interviewer: Who is the one causing these problems?*

*Respondent: It is the government.”*

#### ICDS and MDM

ICDS services are available to all children below 6 years old, and pregnant and lactating women. There was broad awareness of these services, who was entitled to them, and what should be provided. For MDM, which is provided to all children between 6 and 14 years old who attend government schools, there were also high levels of awareness of these services, entitlement and expected provision.

### Navigating access to services

This stage of the candidacy model asks whether an individual has a) knowledge of the existence of services and b) knows how to access them (Dixon-Woods et al., 2005). We asked every respondent whether they were aware of the existence of available NFSA services. Everybody was informed of TPDS. Almost everybody was aware of ICDS and MDM, and most people knew that pregnant women were entitled to assistance (MAMATA), although fewer could identify the details of the scheme. However, knowledge of how to navigate access to these services was more limited, with TPDS and MAMATA considered more ambiguous than ICDS and MDM.

#### TPDS

Navigating access to TPDS services has two levels, navigating the system to access i) a ration card, and ii) subsidised grain.

Navigating the steps necessary to get a ration card was something that some respondents were unable to do, even if they recognised their candidacy, and felt able to assert it and complain, as this 60 year old OBC labourer explains,

*“We are eligible but we have not got that ration card yet… what can we do? We are writing about it… to the Sarpanch [and] the secretary… We can get the rice only if we get the ration card… How else can we get them?”*

Further barriers to navigating this service include incorrect/insufficient adjudication by professionals which are expanded on within stage five below.

All respondents had knowledge of how to navigate TPDS services to access subsidised grain with a ration card (presenting themselves and their ration card at their local Fair Price Shop (FPS)). Without this card, however, it is not possible to navigate this part of the service.

#### MAMATA

Navigating access to MAMATA is the most complex of the four NFSA services. Pregnant women are required to register with the Anganwadi Worker (AWW) and fulfil lengthy criteria to receive two payments (RS 3000 and RS 2000). It is the AWW and Anganwadi Helper’s role to record that women have completed the necessary tasks to receive payment. There were several cases reported of eligible women successfully registered with the AWW, with a MAMATA card, who did not receive payment. The women affected were unable to understand why this had happened, as described by this 35 year old OBC farmer,

“*I: You did not have the instalment yet?*

*R: No.*

*I: Why did not you have it?*

*R: They said that we do not get an instalment in 2 months and 15 days. The passbook did not work.*”

#### MDM and ICDS

Navigating MDM services is relatively easy as it is provided to all school going children in (or nearby) schools. As long as the children attended school, and the service was being correctly provided, there were no navigation problems reported. Similarly, ICDS services were relatively easy to navigate for eligible families. When asked about discrimination or marginalisation on the basis of caste or tribe, a few participants reported that others were not able to access ICDS or education (and therefore MDM) services due to caste or tribal status and there was one report of eggs being withheld.

### Permeability of services

Permeability of NFSA services refers to the demands made by the supply-side gatekeepers and service providers for individuals to show they qualify for a service. Permeability is directly linked to how complex or ambiguous the rules around eligibility are (Dixon-Woods et al., 2005). Complex inclusion criteria for TPDS and MAMATA make these services less permeable than ICDS and MDM.

#### TPDS

The demands made for individuals to show they qualify for TPDS services proved insurmountable for a quarter of respondents who did not have a ration card despite eligibility. Many people were unclear as to why, as described by a 21 year old male OBC student,

“*Interviewer: Why haven’t you claimed [TPDS] before?...*

*Respondent: It is just that we are not getting. Why are we not getting it… I don’t know.*”

#### MAMATA

MAMATA’s long list of demands for qualification prevented access for a few respondents. Pregnant and lactating women aged 19 and over are entitled to receive payments for their first two live births, but they must also register their pregnancy, attend at least two antenatal checks, receive IFA tablets and at least one TT vaccination, attend at least one counselling session, give birth in a government hospital and stay for at least 48 hours.

### Asserting candidacy at services

This stage of the model asks how much work is required by potential service users when interacting with service providers, to obtain access (Dixon-Woods et al., 2005). Asserting candidacy for NFSA services is required at two levels, i) to front-line staff (those providing the service directly to the user) and ii) to gatekeepers and other officials who manage the services. Interacting with front-line staff is relatively simple for all four NFSA services due to the cards provided for TPDS and MAMATA and the clear eligibility guidelines for MDM and ICDS. However, if potential service users are required to interact with individuals responsible for managing or gatekeeping the service (with TPDS and MAMATA) the amount of work required can be high.

#### TPDS

For people without a ration card, not being able to assert candidacy was a significant barrier. Despite being able to access other NFSA services, a 23 year old SC woman has not been able to assert candidacy for TPDS, and is unsure why, despite asking for help from two separate sources. She states,* “I have no ration card… I can’t say [why] but a Panchayat level madam have said they will give ration card..[but] I have no ration card.”*

### Adjudication by professionals.

Adjudication by professionals provides a perspective from the supply side of NFSA. This stage of the model asks whether a service provider or gatekeeper considers an individual legitimate in their claim of candidacy for a service, and then provide it (Dixon-Woods et al., 2005). As described above from the perspective of service users, there are two levels of adjudication by professionals. Front-line service providers are assisted in their adjudication through access cards (TPDS and MAMATA) or clear inclusion guidelines (MDM and ICDS). However, gatekeepers who manage the schemes and are responsible for facilitating access have a more complex role. They were reported to mistakenly, or deliberately, block some users from claiming their eligibility for a service. As the following quote from a female 19 year old SC participant shows,

“*The Sarpanch and Ward member do not listen to our grievances even though we request them [to]… they have obstructed us getting these facilities.*”

#### TPDS

For TPDS services adjudication by front-line professionals is within the community FPS. The experience of professionals reflects that of service users above, the ration card system makes adjudication of candidacy straightforward. In order to be eligible for TPDS a ration card is needed, without one no access is allowed; no problems were reported. However, the second level of adjudication is more complex, and is operationalised when someone has been excluded from TPDS in error. Respondents who do not have a ration card have tried to access them through various gatekeepers, including village leaders (Sarpanch/Ward Members), other NFSA service delivery agents (Anganwadi Workers), or by complaining to village leaders in village level meetings. These individuals often did not recognise eligibility, or if they did they were still not able to achieve access. Several respondents reported that access was being deliberately blocked by certain powerful individuals. This issue is articulated by a 60 year old ST female participant,

“*I have submitted the adhaar [ration] card and all the necessary requirements but they [Sarpanch and Ward Members] don’t seem to take heed of the problem.*”

### Use of or resistance to services

This stage of the model asks whether eligible individuals who are offered access to services decide to use them (Dixon-Woods et al., 2005). Except for a few respondents who were inhibited from access due to geographical distance, no respondents chose not to use NFSA services that were offered. There were some examples of services being considered sub-standard, but they were still used. For example, some TPDS users would have liked increased quantity and/or quality of grains, but they still collected their rations. A few MDM and ICDS users complained similarly of low quantity and quality, but the service was still used. Non-physical components of the service, including counselling and advice, were not mentioned by respondents.

### Local operating conditions

Local operating conditions are factors that influence decisions about service provision and use, such as perceived or actual availability of services and resources, and the relationships that may develop between providers and recipients (Dixon-Woods et al., 2005). This stage incorporates geography, availability and acceptability alongside the links and relationships between the communities and NFSA service providers and gatekeepers, much of which have been discussed above, and are also key here.

The *geographic* distances between some households and NFSA services were a significant barrier for some respondents. In particular the distance to FPS and AWC prevented access. In one respondent’s community, when asked what problems they faced receiving TPDS, a 45 year old male labourer stated,

“*the location of the ration distribution is far away. That is the only problem… they are unable to reach and avail the facilities… The old aged people are unable to walk so far.*”

Another respondent, a 25 year old housewife is unable to send her children to the AWC,

“*The Anganwadi Centre is too far. It becomes problematic for us to go to a distant location for accessing those facilities...*”

A lack of *availability* of services was discussed by over one quarter of respondents as a barrier to access. ICDS, TPDS and MAMATA were all stated to be unavailable to some people, for a variety of reasons. ICDS services were not provided at all in two respondent’s villages as this 54 year old male SC Farmer describes.

“*It's been three years since any of the children of our colony are not getting any ANGANWADI service.*”

There were some complaints raised about the *acceptability* of ICDS, MDM and TPDS provision. ICDS services were considered unacceptable for various reasons including poor quality, not providing the types of food expected (eggs, for example) and the most common issue faced, that the AWW was not providing the requisite amount of food. The complaints of male and female respondents were largely similar, and there was no discernible difference in barriers between tribes and castes.

*“They are supposed to give breakfast in the morning to children, but they don’t do that. Instead, they give meal that is only rice. Also they provide less than what it should be. It is not enough for children to eat.”* *Female, 30, Housewife, OBC*

This was blamed on the AWW themselves in most cases,

“*Anganwadi is not doing its job properly” Female, 23, Farmer, OBC*

and attributed specifically to discrimination against a caste or tribe by that worker in at least two cases.

*“let me tell you what the Anganwadi is doing… When some officer or head comes for some meetings, the Anganwadi workers come to our house to call us to the meetings… they go door to door and tell them to come to the meeting positively… so that there would be no complaint against them… we are unprivileged people… we are not getting food properly or the eggs… they neglect [us].”  Female, 25, Farmer, OBC*

Many respondents do not feel that they are able to complain at all,

“*Interviewer: Are you satisfied with these [Anganwadi] services or not?*

*Respondent: Even if we are not satisfied, what can we really do?*” *Male, 43, Labourer, SC*

Others stated that when they complained nothing changed, creating a negative relationship with service providers and gatekeepers.

## Discussion

Applying the candidacy framework provides a unique perspective of the challenges users of different NFSA services face, going beyond simply whether or not a service was utilised, to uncover why problems with access persist. The candidacy model helps illustrate how barriers to accessing services are more complex than traditional LMIC healthcare access frameworks that concentrate on geography, availability and acceptability (Ensor & Cooper, 2004; Peters et al., 2008; Jacobs et al., 2012), would allow for.

For eligible TPDS service users, there are five potential points within the journey of service use that could prevent access. The complex eligibility criteria and seemingly impenetrable system of entry made *identifying service user candidacy* problematic for both service providers and users. In the case of TPDS, state governments are responsible for identifying beneficiaries. Those deemed eligible are given a ration card by their district food office, which serves as identification for access. However, while technology has been associated in some states with improvements in provision and avoidance of ‘leakages’ (i.e. corruption), though not without some limitations (Joshi et al., 2016), India-wide reforms to digitise entitlements have been controversial, and large numbers of people have been excluded, and included in error (Balani, 2013). Those excluded face a number of hurdles to access, not least identifying whether they are entitled to the service in the first place, followed by navigating a complicated system to attempt to achieve access. Many prospective TPDS service users presumed candidacy for ration cards, but this inability to overcome the entry criteria to the service (*impermeability)* meant confirming their candidacy, and rectifying *incorrect adjudications* was incredibly difficult. This was particularly evident when interacting with gatekeepers and senior officials, rather than front-line staff. *Asserting candidacy* was made relatively easy for those with a ration card, but completely impossible for those without. Such assertions require negotiation with formal or informal service providers (Pradhan & Roy, 2019), but given the already marginalised conditions of these communities and the discrimination traditionally felt by inhabitants of the KBK + districts, particularly if coming from SC and ST groups, such possibilities are already hampered. Other l*ocal conditions*, including geography, and a lack of availability of services also prevent or inhibit access to TPDS.

For eligible MAMATA service users, there are six potential points within the journey of service use that could prevent access. Correctly *identifying candidacy* is complex, with a list of five initial conditions for applying, and more conditions that follow. These conditions were not widely known by respondents. Service providers were not able to easily resolve identification problems. Once candidacy has been identified however, *navigating services* was found to be complex. For example, service users often did not understand what was required to navigate services, for example who was locally responsible for allocating the benefit or who to approach if they had somehow been missed. Similarly to TPDS, those rights holders who were issued with a MAMATA card were easily able to *assert candidacy*. However, access to these cards was problematic due to the *impermeability* of the process; even when both the service user and provider are able to correctly identify candidacy, this step could prove insurmountable. Rectifying *incorrect adjudications* was problematic given the hierarchies that rural women faced in dealing with local officials. *Local conditions* such as the inaccessibility of health or other local government services, particularly to the most marginal ST settlements, also prevented or hampered access to MAMATA.

For ICDS and MDM eligible service users, three possible barrier points along the journey of service were identified. Identifying candidacy for both ICDS and MDM services was relatively straightforward as both services have clear inclusion criteria. The universality of these services (within specific but very clear limits) has made identifying candidacy easier. Universality was a key aim of the reforms that accompanied civil society demands and judicial activism that formed the Right to Food Movement and subsequent NFSA (Sandhu, 2014). High awareness of these services suggests universal roll-out has been partially successful. However, *navigating candidacy* was reported to be problematic for some marginalised people, as a result of their identities. *Resistance to use of services* was reported for both ICDS and MDM due to perceived poor quality and service, although services were still used due to a lack of alternatives. *Local conditions* prevented access to ICDS and MDM services.

The *impermeability* of services, and adjudications made by professionals outlined above were found to be problematic at two specific levels: firstly, front-line staff who deliver services on the ground, and secondly gatekeepers of services, or high-level officials. It became clear that community members experienced broadly better treatment by ground-level service providers, when compared to higher level gatekeepers and officials. This could be because most of the ground-level service providers are part of the same communities they are serving, living in similar conditions within similar geographic locations. Higher level gatekeepers however, were seen as separate from communities, harder to reach, and much less responsive and accountable to communities. Further, many of the aspects of the NFSA are delivered and monitored by community members, creating sometimes unintentional power dynamics within communities.

To identify candidacy for NFSA services individuals either need to recognise a food security need or have knowledge of NFSA inclusion criteria. Recognising a food security need (if not immediately apparent by hunger) will be affected by understandings of food security and nutrition, and therefore education, marginalisation and poverty. Social audits have been introduced within Odisha with the aim of educating marginalised populations about their rights within NFSA programmes, but also, crucially to inform people about mechanisms to hold service providers and gatekeepers accountable. The social audits operate both as an information providing tool, and as a forum for airing grievances, and in some cases immediate redressal is achieved. While it was clear from this research that the problems the social audits aim to fix persist (although this article is not evaluating social audits), the mere fact that these problems have been recognised and a programme to facilitate change has commenced, is a starting point for potential change in Odisha. However, just understanding what one is entitled to (stage 1), and knowing how to access it (stage 2), will not necessarily empower individuals to overcome the barriers created by gatekeepers and high level officials. Using the candidacy model to identify these barriers has helped unpack the complexity and challenges with these processes and can be used to better understand where to target future interventions.

## Conclusion

Using the candidacy model within this LMIC setting has facilitated a broader understanding of the barriers faced by NFSA service users who are from the most marginalised groups in Odisha, highlighting difficulties faced at all seven stages, in an environment without viable alternative options. Interlinked with this are other underlying issues associated with malnutrition, including those identified by Kohli et al. (2017) in Odisha, such as ST/SC and OBC status and associated marginalisation, a lack of women’s empowerment, and a lack of secondary education, which have not been addressed by the state, and inhibit access to services.

The NFSA was already the result of sustained national and state specific advocacy for improved food security that directly influenced the political economy of service delivery nationally (Khera, 2013; GOI, 2013). Empowerment of individuals and communities, through education and specific empowerment programmes (also recommended by Peters et al., 2008; Sharma et al., 2009; Jacobs et al., 2012; Tanksale, 2015) could improve respondents’ ability to identify and assert candidacy, as well as navigate services. This could also lead to increased participation in community mechanisms for accountability and change, such as the social audit process, which have been found to improve both delivery and uptake of NFSA services (Gordon et al., 2019). Improving and building on the existing social audit processes could also lead to improvements from a supply side of NFSA services, including rectifying incorrect adjudications and local conditions.

## Data Availability

The datasets used and analysed during the current study are available from the corresponding author on reasonable request.

## List of abbreviations

APPI: Azim Premji Philanthropic Initiative
FPS: Fair Price Shop
GOI: Government of India
ICDS: Integrated Child Development Services
LMIC: Low and Middle Income Countries
MAMATA: Maternity Benefit Programme (also known as Indira Gandhi Matritva Sahyog Yojana)
MDM: Midday Meal Programme
NFSA: National Food Security Act (India)
NSSO: National Sample Survey Office (India)
OBC: Other Backward Castes
RS: India Rupees
SC: Scheduled Caste
ST: Scheduled Tribe
TPDS: Targeted Public Distribution System
WHO: World Health Organization
